# Nano-Reactors Based on Ovotransferrin Organic Skeleton through a Ferroptosis-like Strategy Efficiently Enhance Antibacterial Activity

**DOI:** 10.3390/jfb15080205

**Published:** 2024-07-24

**Authors:** Zihan Zhuo, Chunfang Yin, Zhenqing Zhang, Yumeng Han, Haoye Teng, Qi Xu, Changming Li

**Affiliations:** 1 Institute of Advanced Cross-Field Science, College of Life Science, Qingdao University, Qingdao 266800, China; zhuo@qdu.edu.cn (Z.Z.); ycf1462722151@163.com (C.Y.); zhangzhenqing0330@163.com (Z.Z.); hanym0105@163.com (Y.H.); hy12122021@163.com (H.T.); 2 Institute of Materials Science and Devices, School of Materials Science and Engineering, Suzhou University of Science and Technology, Suzhou 215009, China

**Keywords:** ovotransferrin, nano reactors, antibacterial, ferroptosis-like death, green synthesis

## Abstract

The issue of bacterial resistance is an escalating problem due to the misuse of antibiotics worldwide. This study introduces a new antibacterial mechanism, the ferroptosis-like death (FLD) of bacteria, and an approach to creating green antibacterial nano-reactors. This innovative method leverages natural iron-containing ovotransferrin (OVT) assembled into an organic skeleton to encapsulate low-concentration adriamycin (ADM) for synthesizing eco-friendly nano-reactors. FLD utilizes the Fenton reaction of reactive oxygen species and ferrous ions to continuously produce ·OH, which can attack the bacterial cell membrane and destroy the cell structure to achieve bacteriostasis. The OVT@ADM nano-reactors are nearly spherical, with an average diameter of 247.23 nm and uniform particle sizing. Vitro simulations showed that Fe^3+^ in OVT@ADM was reduced to Fe^2+^ by glutathione in the bacterial periplasmic space, which made the structure of OVT loose, leading to a sustained slow release of ADM from OVT@ADM. The H_2_O_2_ continuously produced by ADM oxidized Fe^2+^ through the Fenton reaction to produce ·OH and Fe^3+^. The results of the antibacterial assay showed that OVT@ADM had a satisfactory antibacterial effect against *S. aureus*, and the inhibition rate was as high as 99.3%. The cytotoxicity results showed that the mitigation strategy significantly reduced the cytotoxicity caused by ADM. Based on the FLD mechanism, OVT@ADM nano-reactors were evaluated and applied to bacteriostasis. Therefore, the novel antibacterial mechanism and OVT@ADM by the green synthesis method have good application prospects.

## 1. Introduction

Antibiotics have revolutionized medicine, farming, and other fields with their powerful antibacterial effects, but their overuse has led to environmental concerns and an alarming rise in antibiotic-resistant bacteria [1]. This necessitates the development of alternative antimicrobial strategies. Currently, nonantibiotic antibacterials hold considerable promise, but they encounter significant barriers in practical applications due to issues like cytotoxicity, instability, and poor targeting, such as benzalkonium chloride (BKC), which has also been identified as an emerging pollutant with respiratory toxicity and neurotoxicity [2,3,4]. New antibacterial mechanisms are urgently needed to combat resistance and toxicity. Ferroptosis, a new form of iron-dependent cell death characterized by an iron-dependent accumulation of reactive oxygen species (ROS), is biochemically, genetically, and morphologically distinct from apoptosis, necrosis, and cellular autophagy, and it is now being demonstrated to be a potent pathway for killing cancer cells. Although the precise and complete role of iron in ferroptosis remains unclear, the iron-catalyzed ROS production, such as the Fenton reaction, has proven to be an important pathway for iron-induced ferroptosis [5]. At the same time, the onset of ferroptosis is often accompanied by the loss of plasma membrane integrity and the leakage of intracellular contents [6]. The precise mechanisms of ferroptosis are not fully understood, but a critical pathway involves iron-catalyzed ROS production, such as through the Fenton reaction [7,8,9]. The bacterial cell membrane contains unsaturated fatty acids, which play a crucial role in the physiological activity of the cell membrane and are prone to initiating free-radical reactions [10,11,12]. Notably, bacteria with low intracellular reducing substances are more vulnerable to ROS [13]. Based on the material basis and conditions described, this work proposes a novel antibacterial mechanism: the ferroptosis-like death for bacteria (FLD). FLD triggers the Fenton reaction by providing ROS and Fe^3+^, destroying bacterial cell membranes.

Fe^2+^ exhibits dose-dependent cytotoxicity, where excessive levels can induce ROS and increase bacterial lipid peroxidation [14]. A previous study designed iron-loaded proteins loaded with Adriamycin (ADM) for cancer treatment and confirmed that cancer cell death was attracted by ferroptosis [15]. To improve the iron level around the bacteria, we skillfully used the natural iron-containing ovotransferrin (OVT), which is an iron-binding glycoprotein from egg white, with a variety of biological activities such as antibacterial, antioxidant, and other activities [16,17,18]. As the natural substrate of OVT, Fe^3+^ has a strong binding ability with OVT. After binding Fe^3+^, the structure of OVT is closed and tight, and after Fe^3+^ is released, its structure becomes open and loose [16,17,18,19]. When OVT is used as a drug carrier, this property favors the release of drug pH responses in nanoparticles. Glutathione (GSH), a prominent antioxidant in organisms, exhibits diverse biological functions and is crucial in safeguarding cells against oxidative harm and upholding a stable redox milieu. The bacterial periplasm space contains GSH with a reducing effect, which can reduce Fe^3+^ to Fe^2+^, laying the foundation for the occurrence of FLD [20].

In addition, the mechanism of ferroptosis is also affected by the insufficient supply of H_2_O_2_. ADM is an anthracycline antineoplastic drug that has been one of the most effective antineoplastic drugs used alone or in combination with other medications [21]. Low concentrations of ADM can induce the production of H_2_O_2_ [22]. The moiety of anthracene in the chemical structure of ADM can be converted into a semiquinone radical form, and, subsequently, nonenzymatic semiquinone radical can be converted into superoxide (O^2−^) and hydrogen peroxide (H_2_O_2_) by molecular oxygen (O_2_) re-oxidation and interact with various macromolecules [23,24]. Low-concentration ADM could not only compensate for the lack of H_2_O_2_ around the colony but also avoid the strong toxic side effects of ADM. Both OVT and ADM can induce the development of ferroptosis. Nanoparticles built from transferrin and ADM have been shown to induce ferroptosis in cancer cells [15,25,26].

**Scheme 1:**
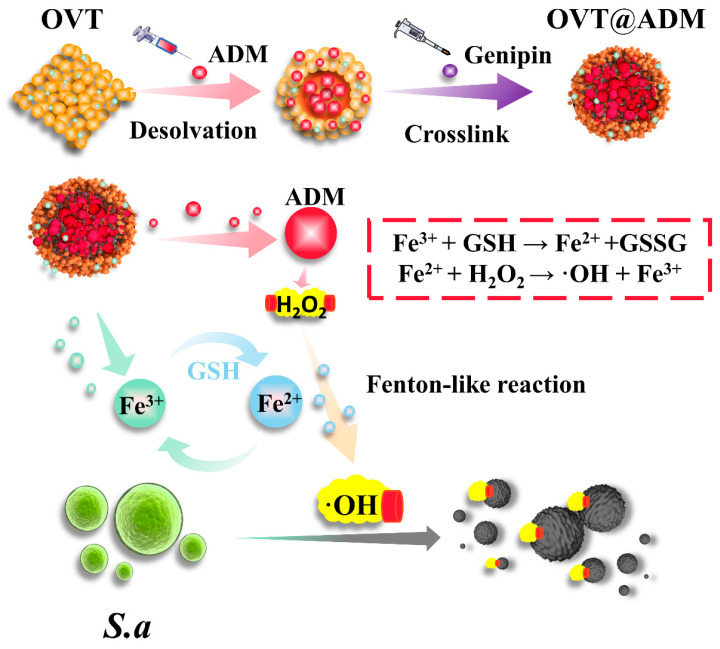
The synthesis process and the antibacterial principle of OVT@ADM. OVT: ovotransferrin; ADM: adriamycin; GSH: glutathione; GSSG: oxidized glutathione; *S. a*: *Staphylococcus aureus*.

Based on the above, we propose to use the FLD mechanism for antibacterial activity in this work. The FLD nano-reactors (OVT@ADM) were constructed here, which loaded ADM on natural iron-containing OVT by the green desolvation method. OVT@ADM nano-reactors did not need to add other auxiliary sterilization methods, such as photothermal and photodynamic treatment, to achieve good antibacterial effects. As shown in Scheme 1, OVT compensated for the lack of iron supply around the bacteria by carrying Fe^3+^ on its own, GSH in the bacterial periplasmic space could reduce Fe^3+^ to Fe^2+^, and low concentrations of ADM were responsible for the production of H_2_O_2_, which underwent a Fenton reaction with Fe^2+^ to produce hydroxyl radicals, leading to FLD causing the bacteria to rupture and, thus, become antibacterial. The OVT@ADM nano-reactors are safe and controllable, could alleviate the adverse effects of antibiotic abuse or toxic agents, and provide an innovative strategy for future antibacterial research.

## 2. Materials and Methods

### 2.1. Materials

The following materials were purchased from commercial suppliers: OVT (extraction from egg white in the laboratory, purity ≥ 95%), ADM, genipin, and methylene blue (MB) were purchased from Macklin (Macklin Biochemical Co., Ltd., Shanghai, China). 1,10-Phenanthroline, Ammonium iron(II) sulfate hexahydrate, Hydroxylammonium chloride, and Glutathione (Reduced) (GSH) were purchased from Aladdin (Shanghai Aladdin Biochemical Technology Co., Ltd., Shanghai, China). OxoidTM yeast extract was obtained from (Thermo Fisher Scientific Co., Ltd., Shanghai, China), Agar from (Beijing Solarbio Science & Technology Co., Ltd., Beijing, China), and trypsin from (Qingdao Hope Bio-Technology Co., Ltd., Qingdao, China). Ultra-pure water was obtained from a Basic2002-UV ultrapure water system (Laibre, Qingdao, China). All other chemicals and solvents used in the study were analytical reagents.

The *Staphylococcus aureus* (ATCC25923) used in this study came from the −80 °C cryopreserved strain of the College of Life Sciences, Qingdao University. The mouse fibroblasts (L929) used in this study were provided by Future Testing Company (Qingdao, China).

### 2.2. Methods

#### 2.2.1. Preparation of Nano-Reactors

OVT-NPs and OVT-ADM were prepared by the desolvent method and by adding genipin crossing [27]. OVT-NPs: OVT was dissolved in ultrapure water and prepared into a 5 mg/mL OVT solution, and insoluble impurities were removed from the filter membrane over 0.45 nm. Absolute ethanol was slowly added to the OVT solution using a syringe, and 5% (*w*/*w*) genipin was crosslinked to a 37 °C water bath and incubated for 4 h [28,29]. The reaction was terminated with glycine, and the ethanol unloaded drug was removed by dialysis.

OVT@ADM: OVT and ADM were dissolved into ultrapure water, respectively, prepared into 5 mg/mL and 3 mg/mL OVT solution and ADM solution, and the insoluble impurities were removed by the 0.45 μm filter membrane. Beyond these differences, the remaining steps align with the method described for OVT-NPs.

#### 2.2.2. Particle Size and Zeta-Potential Measurements

After completing the preparation, the nano-reactors solution was sonicated for 15 s, and 1 mL of the sample was added to the cuvette; the bubbles were driven away and put into the sample pool, the outer cover of the instrument was closed, and the zeta-potential of the nano-reactors was determined. The particle size of nano-reactors was measured based on the principle of dynamic light scattering (DLS) using a nanoparticle-sized zeta-potential meter (Malvern Instruments, Worcestershire, UK). Determination conditions: 3 cycle scans; detection temperature: 25 °C.

#### 2.2.3. Scanning Electron Microscopy (SEM)

The sample solution was lyophilized using a vacuum freeze dryer (ZEISS, Oberkochen, Germany), and a small amount was placed on the silicon wafer. Scanning electron microscopy (Jsm-780of, Nippon Electronics, Tokyo, Japan) was used to detect the surface morphology of lyophilized nano-reactors. All samples were covered with a gold layer before analysis.

#### 2.2.4. Encapsulation Efficiency

First, most of the nano-reactors were separated by high-speed centrifugation (Sk-300, Sigma, Taufkirchen, Germany), and then ultrafiltration was performed at 4000 rpm. The lower solution was detected by a UV absorption value at 478 nm using a UV-spectrophotometer (UV-2600, Shimadzu, Kyoto, Japan) and replaced by the ADM standard curve (R^2^ = 0.9995). The entrapment efficiency and drug loading capacity were calculated. The entrapment efficiency is calculated as follows:

(1) $$\mathrm{Drug}\;\mathrm{loading}\;\mathrm{capacity}\;(\%)=\frac{\left(M_{0}-M_{s}\right)}{M_{f}}\times 100\%,$$

(2) $$\mathrm{Entrapment}\;\mathrm{efficiency}\;(\%)=\frac{\left(M_{0}-M_{s}\right)}{M_{f}}\times 100\%.$$

Here, *M*_0_ refers to the initial weight of ADM, and *M_f_* refers to the total weight of the nano-reactors; *M_S_* refers to the weight of ADM in the supernatant.

#### 2.2.5. UV–Visible Spectroscopy and Fluorescence Analysis

The sample solution was sonicated and dispersed, and then it was added to 2/3 of the height of the cuvette. Then, the sample tank was placed, and the instrument’s outer cover was closed. The UV absorption spectrum of different samples at 350–700 nm was measured by a UV-spectrophotometer (UV-2600, Shimadzu, Japan).

Fluorescence measurements were performed using a fluorescence spectrometer (FS5, Edinburgh Instrument, Livingston, UK). The excitation bandwidth and emission bandwidth were 4.0 nm, and the excitation wavelength was 295 nm. It selectively excites the 214 residue of tryptophan, with an emission wavelength of 330 nm and an emission spectrum recording range of 300~750 nm.

#### 2.2.6. Fourier Transform Infrared Spectroscopy (FTIR) Analysis

The sample solution was lyophilized using a freeze dryer, and the powder was placed on the sample table. Fourier transform infrared spectroscopy (Nicolet is50, Thermo Fisher Scientific Inc., Waltham, MA, USA) was used to obtain OVT-NPs, OVT@ADM, etc., to determine their molecular structure. The samples were scanned 32 times per spectrum.

#### 2.2.7. X-ray Diffraction (XRD) Analysis

The powder X-ray diffraction (XRD) pattern of the samples was investigated (Bruker, Karlsruhe, Germany) using a diffractometer system with Cu-ka radiation in the scan range of 2θ from 10 to 50° and scan rate of 20°/min.

#### 2.2.8. Simulated Release of ADM

In order to study the simulated release in vitro, OVT@ADM solution was reacted under three different conditions: pH 7.4 and GSH (−), pH 5.5 and GSH (−), pH 5.5 and GSH (+). The concentration of Nps was kept at 50 μg/mL, and the concentration of GSH was kept at 0.1 mmol/L. Three groups of mixed solutions were shaken at 5 min at 37 °C and incubated at 37 °C. The release of ADM at different reaction times was recorded by a UV-spectrophotometer.

#### 2.2.9. Reduction of Fe^2+^

OVT@ADM was mixed with GSH mother liquor (10 mM) and shaken for 5 min at 37 °C, and then it was incubated at 37 °C. It was taken out at 0, 30, 60, 90, and 120 min, respectively, and mixed with 0.15% 1,10-Phenanthroline. The UV absorbance of the mixed solution at 510 nm was recorded after a 20 min reaction.

#### 2.2.10. OH Generate

OVT@ADM (50 μg/mL) and GSH mother liquor (2 mM) were mixed and diluted with PBS, shaken well for 5 min at 37 °C, mixed with H_2_O_2_ (100 mM) and MB (100 μg/mL), and incubated at 37 °C, and the UV absorption spectra of the mixed solution at different times were recorded. At the same time, a group of the mixed solution without GSH (PBS instead of GSH solution) was set up, where the other conditions were the same; it was incubated at 37 °C, and the UV absorption spectra of the mixed solution at different times were recorded.

#### 2.2.11. Antibacterial Property Test

The antibacterial effect of OVT@ADM was evaluated by plate coating using *S. aureus* (ATCC25923) grown in the LB medium. In this study, a diluted bacterial suspension (10^7^ CFU/mL) was mixed with ultrapure water (control), OVT-NPs, free ADM, and OVT@ADM, respectively. The concentration of OVT and OVT@ADM was 1 mg/mL and 1.02 mg/mL, respectively. The concentration of ADM was 18.5 and 8 μg/mL, and it was cultured in an incubator at 37 °C. Then, 100 μL of diluted bacterial solution was coated on the petri dish with an LB medium and incubated at 37 °C for 24 h. Here, we photographed plots of colonies on the dish.

In this study, we also observed the effect of no sample treatment on bacterial morphology by SEM. Firstly, the bacteria treated with ultra-pure water, free ADM, and OVT@ADM were collected by centrifugation, and the mixture was fixed overnight with glutaraldehyde at 4 °C. After fixation, the mixture was washed with ultrapure water, and then the bacteria were gradually dehydrated by successively treating them with a series of concentration gradients of ethanol. Finally, the bacterial solution was coated on the silicon dioxide wafer, dried, and sprayed with a gold layer, which was observed by a scanning electron microscope [30].

#### 2.2.12. Cytotoxicity Assay

The L929 cell survival assay was used to test the toxicity of OVT@ADM. When the confluence of L929 cells in the culture bottle reached 80%, cells were digested with 0.25% trypsin and prepared as a cell suspension. Cells were counted, seeded at 6000 cells/well in 96-well cell culture plates, and incubated in a 37 °C cell incubator for 24 h. The 100 μL sample was mixed with a 900 μL culture medium, and the old medium in the 96-well plate was discarded. A volume of 300 μL of the mixture was added to each well, and 3 multiple holes were set up for each sample; the culture was continued for 24 h in a cell incubator at 37 °C. In 96-well plates, 100 μL of a new medium and 10 μL CCK-8 solution were added per well for 1–4 h for about 20 min to observe the degree of color development, and OD was read at 450 nm and 630 nm using a microplate reader.

## 3. Results and Discussion

### 3.1. Preparation and Characterization of the Nano-Reactors

#### 3.1.1. Green Synthesis of Nano-Reactors

The OVT@ADM nano-reactors were constructed using renewable raw materials: leveraging OVT from egg white as the organic skeleton and genipin from gardenia fruit as the cross-linking agent to load ADM [16,31]. The synthesis process only involves ethanol, water, and glycine. The nanoparticles produced by this method do not generate heavy metal pollution, do not persist in the environment for a long time, and do not cause severe pollution to nature, thus achieving green synthesis.

#### 3.1.2. Particle Size and Zeta-Potential Measurements

**Figure 1:**
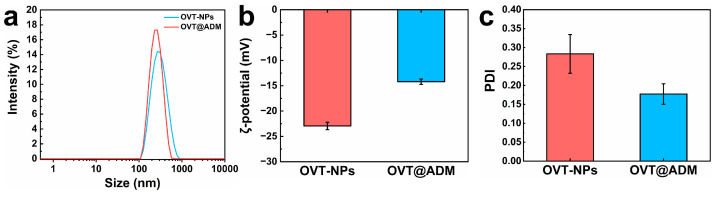
Particle size (**a**), zeta-potential (**b**), and PDI (**c**) of OVT-NPs and OVT@ADM.

The mean hydration size and polymer dispersity index (PDI) of OVT-NPs, OVT@ADM are shown in Figure 1 and Table 1. From the particle size test results, the particle size of OVT-NPs was 256.57 ± 6.42 nm, but when ADM was loaded into OVT, the hydrodynamic diameter of OVT@ADM nano-reactors was slightly reduced to 247.23 ± 4.37 nm. The results show that the intermolecular cross-linking of OVT could prepare monodisperse nano-reactors, and the loading of ADM could make the OVT nanoparticles more compact. PDI was an important index to evaluate the size uniformity of nanoparticles [32]. The smaller the value, the more uniform the size distribution of nanoparticles. As shown in Table 1, the PDI was 0.283 ± 0.051 for OVT-NPs and the PDI for OVT@ADM was 0.223 ± 0.018, both with small PDI values, indicating the same size distribution of the nano-reactors. Zeta-potential is the electrostatic repulsion existing in the surface charge of suspended particles, which is an important index to evaluate the stability of particle suspension [33,34]. To investigate the changes in the zeta-potential of nano-reactors after ADM loading, the zeta-potentials of the OVT-NPs and OVT@ADM were tested. Due to the deprotonation of carboxyl groups, OVT has a negative charge in the range of pH 9–6.218. As shown in Table 1, OVT had a negative charge, and the potential of OVT-NPs and OVT@ADM was negative. After loading ADM, the potential of the OVT@ADM nano-reactors changed from −22.94 mV to −14.21 mV. This phenomenon might be caused by the attachment of ADM to the surface of nano-reactors rather than being completely encapsulated in NPs, and the interaction of OVT with genipin neutralizes some of the amino groups that also have an effect on it. As shown in Table 1, what is noteworthy is that the entrapment efficiency of ADM was 86.7%, indicating a strong OVT drug loading capacity. The results show that OVT@ADM had a narrow particle size distribution, high zeta-potential, and good entrapment efficiency, which provide conditions for its excellent bacteriostatic effect.

#### 3.1.3. Scanning Electron Microscopy (SEM)

**Figure 2:**
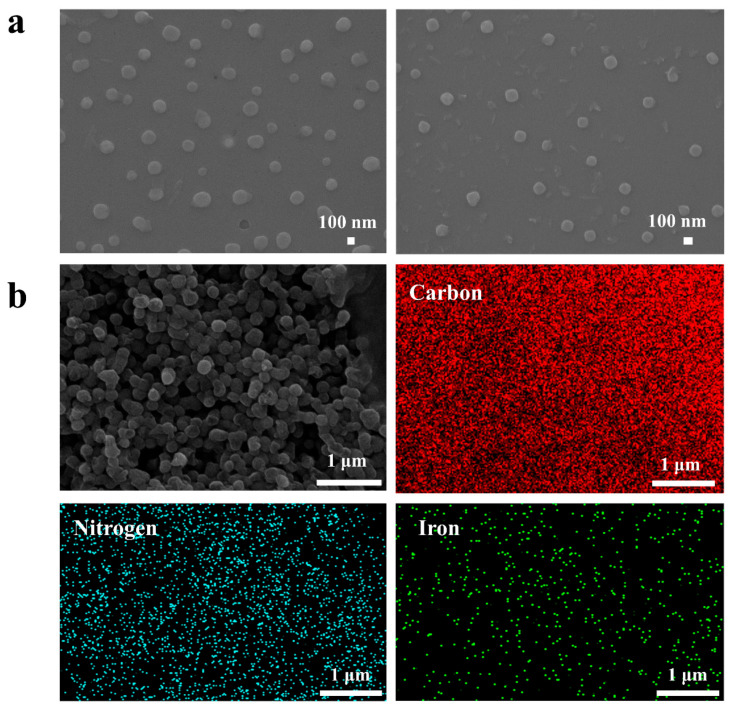
(**a**) FE-SEM images of OVT-NPs (left) and OVT@ADM (right), scale = 100 nm. (**b**) EDS of OVT@ADM, scale = 1 μm.

In Figure 2a, the morphology of the OVT-NPs and OVT@ADM nano-reactors are provided by field emission scanning microscopy (FE-SEM). Consistent with the particle size analysis, both particles show an approximate spherical morphology and similar dimensions. The OVT@ADM was regularly spherical and evenly dispersed. After loading ADM, the morphology of protein nanoparticles did not change significantly, indicating that the loading of ADM had no significant effect on the morphology of protein nanoparticles. Considering the shelf life, microbial contamination, convenience, and transportation cost, freeze-dried powder has advantages in transportation, storage, and distribution. Therefore, the freeze-drying of nanoparticles is very important in practical applications [35]. The morphology of freeze-dried OVT@ADM was tested here. As shown in Figure 2b, OVT@ADM maintained its physical properties in the drying process, indicating that it had good stability. At the same time, we also measured the iron on OVT@ADM by the EDS spectrum. As shown in Figure 2b (iron), after the formation of OVT@ADM nano-reactors by OVT, iron remained uniformly distributed on the surface of the OVT@ADM nano-reactors, which provided the conditions for the Fenton reaction to proceed.

### 3.2. Structural Properties

#### 3.2.1. UV–Visible Spectroscopy and Molecular Fluorescence Analysis

**Figure 3:**
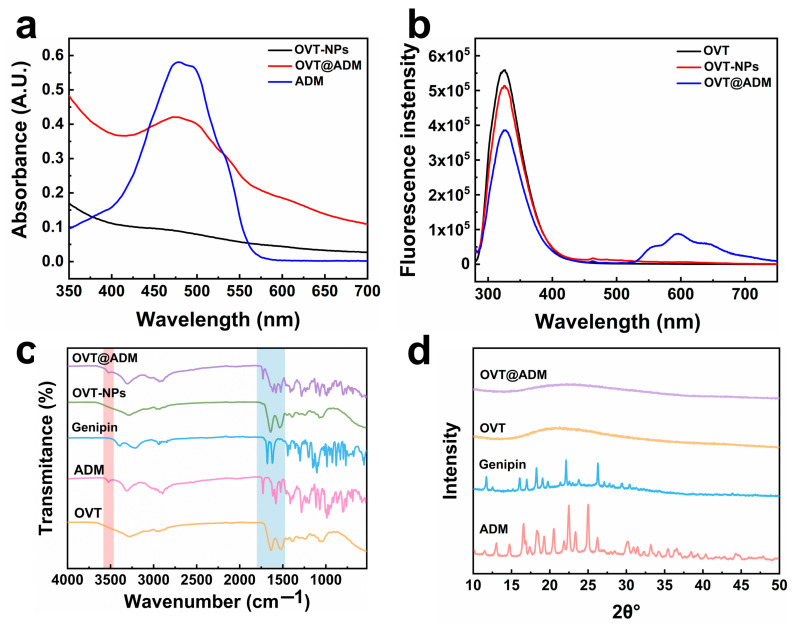
UV–Vis absorption spectra (**a**), fluorescence spectrogram (**b**), FT-IR spectra (**c**), and XRD patterns (**d**) of different samples.

As shown by the UV–Vis absorption spectra (Figure 3a), both ADM and OVT@ADM had UV absorption characteristic peaks at 478 nm, while OVT-NPs had no absorption peak there, which proved that ADM had been successfully loaded into the OVT@ADM nano-reactors. Fluorescence spectroscopy is an effective method to explore the interaction between small molecules and macromolecules such as proteins, which provides valuable information for their binding mechanism. The inherent fluorescence of proteins may be caused by three aromatic amino acid residues—tryptophan, tyrosine, and phenylalanine19. It can be seen from Figure 3b that both free OVT and OVT@ADM had characteristic peaks at 330 nm. When free OVT was changed to OVT-NPs, the intensity of the fluorescence emission peak at 330 nm decreased, but the shape of the peak remained unchanged, showing a typical fluorescence-quenching phenomenon. When ADM was encapsulated in OVT@ADM nano-reactors, the intensity of the fluorescence emission peak at 330 nm was further weakened, and the shape of the peak remained unchanged, indicating that there was an interaction between ADM and OVT. As can be seen in Figure 3a,b, ADM was successfully loaded into the OVT@ADM nano-reactors and caused a change in the tertiary structure of the protein.

#### 3.2.2. Fourier Transform Infrared Spectroscopy (FTIR) and X-ray Diffraction (XRD) Analysis

To evaluate the effect of ADM addition on the OVT@ADM nano-reactors, the FTIR spectra of different samples were compared. The amide I region was more suitable for determining the secondary structure of the protein than the other amide regions because compared to the other amide regions, its vibration comes from an amide functional group [36]. As shown in Figure 3c, the absorption peak of OVT was at 1644.16 cm^−1^, which represents the amide I region formed by the stretching vibration of the C=O stretching of the amide group and the action of a part of C-N [37]. After free OVT was transformed into OVT-NPs, the absorption peak changed from 1644.16 cm^−1^ to 1640.39 cm^−1^. And the peak intensities of α-helix (1651 cm^−1^) and β-turns (1669 cm^−1^) decreased, indicating that the secondary structure of OVT changed [37]. After the nano-reactors were loaded with ADM, the absorption peak was at 1615.91 cm^−1^, indicating that the secondary structure of OVT had further changed after the formation of nanoparticles. ADM had a characteristic absorption peak at 3526.46 cm^−1^, attributed to N-H expansion vibration, and this characteristic peak also existed in OVT@ADM and was blue-shifted to 3522.67 cm^−1^, proving that ADM was successfully loaded on OVT@ADM nano-reactors.

Figure 3d shows the X-ray diffraction spectra (X-ray diffraction profile) of pure OVT, pure ADM, genipin, and OVT@ADM at 2θ at 10–50. It can be observed that there were many obvious reflections in the diffraction patterns of ADM and genipin, indicating that they had crystalline properties. In contrast, OVT was a colloidal material that exhibited patterns of amorphous polymers. As shown in Figure 3d, the distinctive sharp reflections of ADM and genipin disappeared in the XRD patterns of OVT@ADM.

### 3.3. In Vitro Release Analysis

#### 3.3.1. Simulated Release of ADM

**Figure 4:**
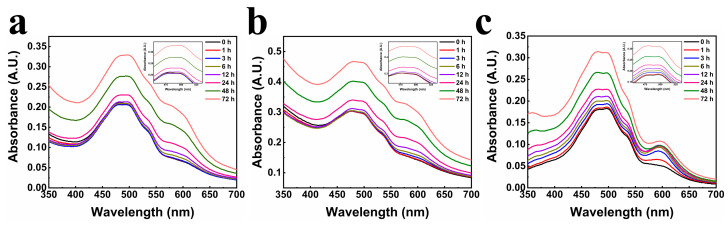
The in vitro drug release curve of OVT@ADM under the condition of pH 7 (**a**), pH 5.5 (**b**), and pH 5.5 + GSH (**c**).

H_2_O_2_ production was controlled by ADM, and a low concentration of ADM can effectively produce H_2_O_2_, while a high concentration of ADM will lead to high cytotoxicity, so the release of ADM is very important. As can be seen from Figure 4a,b, the changes in pH alone had no significant effect on the release of ADM, and there was almost no release of ADM in the first 12 h. However, after adding GSH under acidic conditions, 5% of ADM was released within 12 h and 8% of ADM was released within 24 h (Figure 4c), which is equivalent to twice the release rate of the control group (Figure 4a). Within 72 h, three groups of different treatments of OVT@ADM released 55% of ADM. As shown in Figure 4c, the release efficiency of ADM was significantly increased by the combined effect of acidic conditions and GSH compared with the other two groups.

A total of 46% of ADM was loaded on the OVT@ADM surface, which made the surface potential of OVT@ADM lower than that of the nanoparticles without ADM, which is consistent with the result of the zeta-potential of OVT@ADM being lower than that of OVT-NPs in Figure 1b. This is because in a neutral environment, the nucleophilic amino group (nucleophilic amino groups) in OVT attacked olefinic carbon atom at C-3 in genipin of the olefin carbon atom of genipin at C-3, opened the dihydropyran ring dihydropyran ring, and made the ester group in the genipin replace nucleophilic substitution, forming a secondary amide secondary amide with OVT [29]. ADM also contained the amino group, which reacted with the olefinic carbon atoms at the C-3 position of genipin, and could also cross-link with genipin. Therefore, part of the ADM was cross-linked on the surface or inside of the OVT@ADM nano-reactors, and part of the ADM was not cross-linked and was wrapped in the OVT@ADM nano-reactors due to the protein tertiary structure. When the OVT structure became loose, the uncross-linked ADM was released, while the ADM cross-linked to the interior could not be released rapidly. The results show that ADM had a slow-release effect, which both avoided the rapid drug depletion due to burst release and provided a low concentration of ADM to compensate for the insufficient supply of H_2_O_2_.

#### 3.3.2. Reduction of Fe^2+^

**Figure 5:**
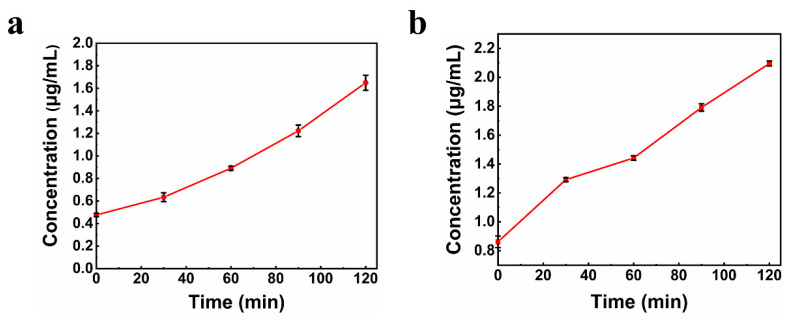
The Fe^2+^ release curves of OVT-NPs (**a**) and OVT@ADM (**b**) in the external environment at 37 °C.

The existence of Fe^2+^ is very important in the process of FLD, and it participates in the production of ·OH, which is essential for inducing FLD. Phenanthrolines is a class of multifunctional chelating agents, which are well known for their formation of non-fluorescent complexes with Fe^2+^, and it is very important in coordination chemistry. 1,10-Phenanthroline is the parent compound of the phenanthroline [38]. 1,10-Phenanthroline and its derivatives have been widely used as analytical probes [39]. 1,10-Phenanthroline reacted with Fe^2+^ to produce an orange-red complex with a maximum absorption peak at 510 nm without such a complex with Fe^3+^ [40]. GSH could reduce Fe^3+^ to Fe^2+^, and Fe^2+^ reacted with 1,10-Phenanthroline to form an orange-red complex. After the reaction of the sample with GSH for a period of time, the mixed solution was mixed with 1,10-Phenanthroline, and the absorbance value of the product at 510 nm was measured. Figure 5 shows the concentration change curve of Fe^2+^ at different times. According to Figure 5a, the Fe^2+^ concentration of OVT-NPs in 120 min increased from 0.476 μg/mL to 1.65 μg/mL, and the Fe^2+^ concentration increased by 247% (1.2 μg/mL). The UV absorption characteristic peak of ADM was at 478 nm, which is close to the UV absorption characteristic peak of the orange-red complex, and had influence on its UV absorption value. But as shown in Figure 4, ADM was hardly released within 120 min, so the rise in UV absorption values at 510 nm is attributed to the generation of Fe^2+^; the Fe^2+^ concentration of OVT@ADM was increased by 144% (1.25 μg/mL). The Fe^2+^ concentrations of OVT-NPs and OVT@ADM both increased by about 1.2 μg/mL within 120 min, indicating that the addition of ADM would not have a significant impact on the production and release of Fe^2+^. The Fe^3+^ carried in OVT could be successfully reduced to Fe^2+^ by GSH, which provides a prerequisite for the smooth progress of the Fenton reaction.

#### 3.3.3. ·OH Generation

**Figure 6:**
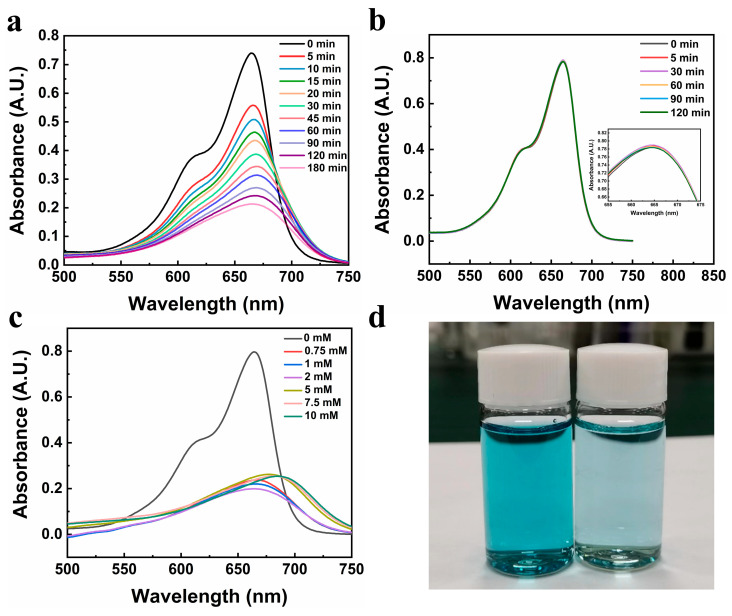
(**a**) Changes in MB uptake at different time points in solutions containing OVT@ADM (50 μg/mL), GSH (0.1 mM), and H_2_O_2_ (25 mM). (**b**) Changes in MB absorption at different time points in solutions containing OVT@ADM (50 μg/mL) and H_2_O_2_ (25 mM). (**c**) In the presence of H_2_O_2_ (20 mmol/L), the effect of different GSH concentrations on the absorption peak of MB. (**d**) Intuitive diagram of adding MB to the mixed solution; no GSH was added on the left, and GSH was added to the right to react. MB: Methylene blue; GSH: Glutathione.

Hydroxyl radicals (·OH) play an important role in the process of FLD. It is mainly produced by the Fenton reaction between H_2_O_2_ and Fe^2+^, which further induces FLD. H_2_O_2_ could be converted to more toxic hydroxyl radicals by undergoing a Fenton reaction with Fe^2+^. To prove that OVT@ADM had the ability to produce ·OH, we used a specific indicator of ·OH: methylene blue (MB) [41]. MB showed strong absorption at 664 nm and could bleach in the action of ·OH. As shown in Figure 6a, the typical absorption of MB was gradually decreased after incubation of OVT@ADM in solutions containing H_2_O_2_ and GSH, indicating that OVT@ADM exhibited good ·OH generation capacity. In the group without GSH reaction, the absorption of MB did not decrease, indicating that without the participation of GSH, Fe^3+^ could not be transformed into Fe^2+^, and ·OH could not be produced by a Fenton reaction, resulting in MB not being degraded (Figure 6b). As shown in Figure 6d, the addition of GSH prompted a Fenton reaction that could significantly degrade MB and lead to MB discoloration compared to the system without GSH addition.

Since GSH had antioxidant effects that inhibited the occurrence of FLD, the effect of GSH concentration in the periplasmic space on ·OH production was evaluated here [42]. The Fe^3+^ contained in OVT@ADM could effectively reduce the concentration of GSH and make the bacteria more sensitive to FLD. As shown in Figure 6c, the typical absorption peak of MB decreased with the increase in GSH concentration in the system. When the GSH concentration reached 5 mM, the characteristic absorption peak increased slightly, but it was still significantly lower than that of the control group. Moreover, when the concentration of GSH was as high as 10 mM, the characteristic peak of MB was still significantly decreased, indicating that a high concentration of GSH could be reduced to generate more Fe^2+^ and then generate ·OH in a Fenton reaction with H_2_O_2_ to degrade more MB. When the concentration of GSH increased to 10 mM, MB could still be degraded effectively, indicating that even if there was a high concentration of GSH, the system could still produce ·OH efficiently. The above results show that OVT@ADM could effectively produce ·OH after reducing Fe^3+^ to Fe^2+^ by GSH. This laid a foundation for inducing FLD.

### 3.4. Anti-Bacterial Properties

#### 3.4.1. The Antibacterial Properties

**Figure 7:**
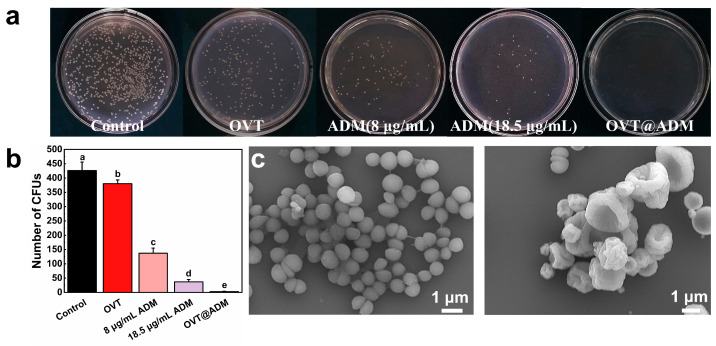
(**a**) Representative images of *S. aureus* grown on different samples after 24 h of culture; from left to right: control group, OVT group, ADM group (8 μg/mL), ADM group (18.5 μg/mL), and OVT@ADM group. (**b**) *S. aureus* colonies on different samples were cultured for 24h and counted. (**c**) SEM images of *S. aureus* under normal conditions (left) and treated with OVT@ADM (right). Letters (a–e) that are different in this figure indicate the significant difference between different series of concentration levels (*p* < 0.001).

The results of the above in vitro simulations show that OVT@ADM could successfully perform the Fenton reaction. Here, we used the plate coating method to analyze the antibacterial activity of OVT@ADM. The number of colony-forming units (CFUs) is shown in Figure 7b. The control group was *S. aureus* incubated with ultrapure water, and the control group had dense colonies with a colony count of 296, indicating that *S. aureus* grew well on the LB medium. There was no obvious difference between the OVT-NPs group and the control group. The colony number was 292, and the concentration of the OVT-NPs group was much lower than its minimum inhibitory concentration, so it did not show an antibacterial effect. The results of the in vitro release experiments showed that ADM was not released from OVT@ADM during incubation time (Figure 4); 30% of the loaded ADM existed on the surface of the OVT@ADM nano-reactors, and the rest was wrapped inside the OVT@ADM nano-reactors. Therefore, two different concentrations of pure ADM were set up: the concentration of ADM loaded on the surface of the OVT@ADM nano-reactors (8 μg/mL) and the concentration of all ADM loaded on the OVT@ADM nano-reactors (18.5 μg/mL). The colony number of the pure ADM group (8 μg/mL and 18.5 μg/mL) was 137 and 38, respectively, indicating that ADM itself had a certain antibacterial effect. It is worth noting that the antibacterial effect of OVT@ADM was very obvious, only two colonies were found, and the inhibition rate was up to 99.3%. This indicates that the ADM in the OVT@ADM nano-reactors, although mostly encapsulated inside the nano-reactors and unable to be released, was conducive to its rapid Fenton reaction with Fe^2+^ in the system to produce ·OH. The antibacterial ability of OVT@ADM is attributed to the Fenton reaction of iron carried in OVT with H_2_O_2_ produced by ADM to produce ·OH, and a portion of ADM’s own antibacterial ability, both of which acted synergistically to exert antibacterial effects.

After *S. aureus* was fixed with glutaraldehyde and dehydrated from ethanol, the surface morphology was observed using scanning electron microscopy. According to Figure 4c, the surface of normal *S. aureus* was smooth, and *S. aureus* was uniformly distributed spherically. After OVT@ADM, the morphology of *S. aureus* changed significantly, the cell surface became rough, the cells were deformed, and the surface underwent shrinkage. This might be caused by a distortion of the cell membrane as well as a leakage of intracellular material [43]. From this, we infer that the antibacterial activity of OVT@ADM was synergized by the antibacterial activity of ADM itself as well as by FLD. Among these, the antibacterial mechanism of FLD was cellular damage caused by an imbalance between reactive oxygen species and the antioxidant system within the biomolecule. FLD killed cells through iron-dependent accumulation of ·OH and could reduce the concentration of GSH in bacteria, making the bacteria less antioxidant and more susceptible to attack by ·OH. At the same time, OVT@ADM killed the bacteria by disrupting the cell membrane of *S. aureus*, causing cellular crumpling and possibly leading to leakage of cellular contents. This shows that FLD is expected to be an effective mechanism for killing Gram-positive bacteria in the future.

#### 3.4.2. Cytotoxicity Analysis

**Figure 8:**
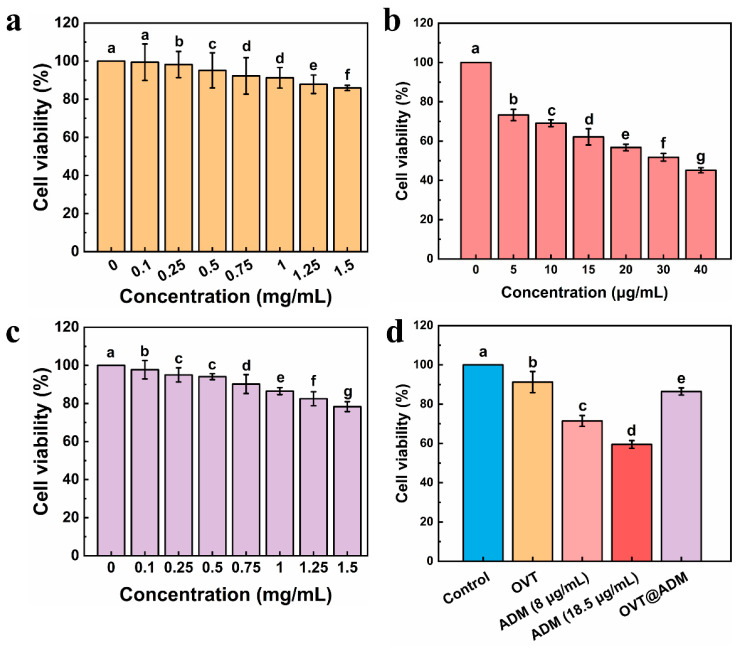
The cell viability of L929 cells treated with different concentrations of (**a**) OVT, (**b**) ADM, (**c**) OVT@ADM, and (**d**) samples used in the bacteriostatic experiment. Letters (a–g) that are different in this figure indicate the significant difference between different series of concentration levels (*p* < 0.05).

As shown in Figure 8a–c, there was a dose-dependent cytotoxicity of OVT, ADM, and OVT@ADM on L929 cells at all concentrations tested. The cell viability after a low concentration of OVT treatment was greater than 90%, indicating low cytotoxicity. When the concentration reached 1.5 mg/mL, the cell viability was still maintained at about 88%, indicating the low toxicity of OVT to L929 cells. However, the cell viability decreased to 70% after treatment with a low concentration of ADM (5 μg/mL) and was only 50% when the concentration was increased to 40 μg/mL, indicating that ADM was strongly cytotoxic. As shown in Figure 8c, when ADM was loaded into the OVT@ADM nano-reactors, cell viability was significantly increased after a series of concentration treatments, indicating that loading ADM into the nano-reactors could significantly reduce the cytotoxicity of ADM. This may be related to the incomplete release.

Further, the cytotoxicity of OVT@ADM at antibacterial concentrations and its corresponding concentrations of free OVT and free ADM were evaluated. Since ADM was not released during the incubation time of the antimicrobial assay, the cytotoxicity of free ADM equivalent to the concentration of ADM loaded on the surface of 1 mg/mL OVT@ADM nano-reactors (8 μg/mL) and the concentration of free ADM loaded in the OVT@ADM nano-reactors in its total concentration (18.5 μg/mL) were compared here. As shown in Figure 8d, the cell viability decreased to 71.5% and 59.5% after 8 μg/mL and 18.5 μg/mL ADM treatment, respectively, indicating that ADM had strong cytotoxicity at this concentration. The cell viability increased to 86.47% after treatment with the same concentration of OVT@ADM (1 mg/mL), indicating that OVT@ADM could effectively reduce the cytotoxicity caused by ADM. Therefore, the OVT@ADM nano-reactors could significantly reduce the cytotoxicity of the drug while exerting its good antibacterial effect. This indicates that OVT@ADM had significantly enhanced antibacterial activity and high security as a practical biomaterial.

## 4. Conclusions

In conclusion, OVT@ADM nano-reactors were synthesized through green methods using renewable resources. The experiments proved that OVT@ADM had excellent antibacterial effect and could significantly reduce the cytotoxicity of ADM. By using scanning electron microscopy, the OVT@ADM nano-reactors were found to be spherical; the average hydrodynamic diameter of OVT@ADM was 247.23 ± 4.37 nm, and it had a small PDI and a zeta-potential of −14.21 ± 0.533 mV. The material has good stability and prospects for application in different systems. The zeta-potential showed that OVT@ADM could successfully convert Fe^3+^ to Fe^2+^ in the presence of GSH and further reacted with H_2_O_2_ produced by ADM to generate ·OH, which eventually induced FLD and caused the bacteria to crumple and die. Notably, the OVT@ADM nano-reactors exhibited favorable biocompatibility and maintained robust antibacterial activity, underscoring their potential for medical applications. Importantly, the OVT@ADM nano-reactors represent a novel, green, and eco-friendly approach to antimicrobial strategies. The FLD mechanism exerts its antibacterial effects, showing potential for infection control and the creation of sterile cell systems. This discovery introduces a pioneering approach to the development of sustainable bacteriostatic materials, offering a fresh perspective for future research in the field of environmentally benign antimicrobial agents.

## Figures and Tables

**Table 1:** Characteristic data of OVT-NPs and OVT@ADM.

Sample	Size (nm)	Zeta-Potential (mV)	PDI	EE (%)
OVT-NPs	256.57 ± 6.42	−22.94 ± 0.73	0.283 ± 0.051	-
OVT@ADM	247.23 ± 4.37	−14.21 ± 0.533	0.223 ± 0.018	86.7

PDI: Polymer dispersity index; EE: Entrapment efficiency.

## Data Availability

The data presented in this study are available on request from the corresponding author.

## Abbreviations

Abbreviation	Meaning
FLD	ferroptosis-like death
BKC	benzalkonium chloride
OVT	ovotransferrin
ADM	adriamycin
ROS	reactive oxygen species
GSH	glutathione
GSSG	oxidized glutathione
*S. a*	*Staphylococcus aureus*
NP	nanoparticle
SEM	scanning electron microscopy
FTIR	Fourier transform infrared spectroscopy
XRD	X-ray diffraction
PDI	Polydispersity
MB	methylene blue
CFU	colony forming units
